# Possible influence of reflecting on the tense of the client's utterances: A preliminary finding

**DOI:** 10.1002/pcn5.70407

**Published:** 2026-09-07

**Authors:** Tomoyuki Sugita, Hiromi Tsujii, Takao Iwanami, Suimei Morikawa, Tadafumi Kato

**Affiliations:** ^1^ Department of Psychiatry and Behavioral Science Juntendo University Graduate School of Medicine Tokyo Japan; ^2^ Yuurin Clinic Tokyo Japan

**Keywords:** mental health, open dialogue, psychotherapy, reflecting team, verb tense

## Abstract

**Aim:**

Open Dialogue (OD), developed in Finland, has gained attention in psychiatric care for its emphasis on human rights. However, quantitative research remains limited, and its implementation in Japan is still at an early stage. Reflecting, originally developed in Norway, is a core element of OD's dialogical practice and has shown promise, yet studies on its mechanisms remain scarce.

**Methods:**

This study examined how reflecting is associated with changes in the tense structure of client utterances. Audio from outpatient sessions was transcribed, and utterances were categorized into four tense types: future, present, recent past, and past. Changes before and after reflecting were analyzed using repeated measures ANOVA and Wilcoxon tests.

**Results:**

Results showed that past‐oriented utterances significantly increased in the reflecting group, while no significant changes occurred in the individual consultation group.

**Conclusion:**

These findings suggest that reflecting may be associated with increased references to past experiences, which may be related to self‐reflection as one aspect of a deepening dialogical process.

## INTRODUCTION

Psychiatric treatment has been using problem‐solving approaches aiming at relief of symptoms. In recent years, however, more holistic support to achieve recovery from illness emphasizing the respect for human rights has garnered increasing attention.

The World Health Organization's (WHO) *Guidance on Community Mental Health Services* (2021) highlighted the importance of human rights and recovery‐oriented care. Among the approaches introduced as “good practices” in this guidance is Open Dialogue (OD), a dialogical approach practiced in Finland.^1^

OD has been reported to reduce the need for inpatient treatment for schizophrenia in Western Lapland.^2^ Additionally, a retrospective cohort study conducted in Denmark found that OD was associated with a decrease in acute psychiatric consultations.^3^ However, few studies have quantitatively evaluated the effectiveness of OD, and existing studies have been criticized for significant bias, suggesting the need for further research.^4^

In Japan as well, interest in OD is growing, and efforts toward implementation in practice are gradually increasing. Nevertheless, quantitative studies on OD remain limited, and its clinical application is still in an early stage. OD is an approach deeply connected not only to therapeutic conversations but also to the mental health system of the community in which it is practiced. As Seikkula and colleagues described, it is supported by institutional elements referred to as “micropolitics”.^5^ Since, the mental health system in Finland, where OD is implemented, is vastly different from that of Japan, the direct adoption of all elements of OD as practiced in Finland is difficult. Yahara (2023) stated that “transplanting all elements of Finland's approach into Japan, which has entirely different contextual conditions, is not only difficult but also not necessarily effective”.^6^ This situation may also be applicable to other countries having medical systems different from those in Finland.

As outlined in “The Twelve Key Elements of Fidelity to Dialogic Practice in Open Dialogue”, proposed by Mary Olson and Jaakko Seikkula as essential components for practicing dialogic therapy,^7^ one of the core elements in OD's dialogical practice is “reflecting”. A typical form of reflecting involves mental health professionals listening to the narratives of the client and their family during a session and then openly discussing their impressions and thoughts in front of them. This method was proposed by Andersen in Norway in 1985 as a reversal of the traditional one‐way mirror technique used in family therapy. It began as a practice in which conversations previously held behind closed doors were made visible to the client and family.^8^ By re‐examining the fixed hierarchy where professionals unilaterally observe and guide clients toward specific solutions, reflecting opened the way for both clients and therapists to gain new perspectives and understandings, potentially changing how problems were perceived and addressed.^6^

Balcells‐Olivero et al. reported that reflecting may also be useful for acute psychiatric inpatients. In their study, they implemented reflecting six times during inpatient care in a psychiatric acute ward for six patients with coexisting psychiatric and substance use disorders. The intervention involved the patients themselves, physicians, and professionals involved during hospitalization (such as nurses, residents, and social workers). Although one participant died by suicide four months after discharge, the study reported a significant reduction in the number and duration of psychiatric hospitalizations in the six months after discharge compared to the six months prior.^9^ Despite such findings, research on the clinical application of reflecting remains limited.

Harris and Crossley (2021) conducted a systematic review of 11 qualitative studies related to reflecting, summarizing their effects from the client's perspective. While clients initially felt confused by the unfamiliar format, they gradually adapted to the unique style of dialogue and reported developing new perspectives and feeling respected.^10^ However, all 11 studies reviewed were qualitative, and no quantitative evaluation was included. To the best of our knowledge, there has been no quantitative study trying to elucidate the reasons why reflecting is effective.

Anderson, the founder of reflecting, stated that clients begin an internal dialogue with themselves while listening to the conversations between others. This internal dialogue then influences how they engage in subsequent conversations with others. Such reciprocal interactions between one's inner thoughts and external dialogues may help to foster new insights for both clients and therapists, opening up possibilities for discovering perspectives beyond conventional understanding.^11^, ^12^

In our clinical practice using reflecting, we have observed that the dialogue tended to deepen in diverse ways. From this experience, we hypothesized that one aspect of this deepening may be measured by examining the effect of reflecting on the change in the tense of utterances during conversations. This study was conducted to test this hypothesis.

## METHODS

### Clinical background of the clinic

#### Overview

This study is not an interventional study but an observational study that was conducted within the framework of the routine medical consultations at Yuurin Clinic. At Yuurin Clinic, during the initial outpatient consultation, clients are verbally asked whether they prefer “a consultation with a psychiatrist and family therapist, including reflecting” (hereafter referred to as a “reflecting consultation”) or “a consultation conducted solely by a psychiatrist” (hereafter referred to as an “individual consultation”). Based on their preference, one of these two consultation formats is provided. Clients are also informed that they can switch between consultation types at any time during the treatment. In cases where the client is experiencing severe depressive symptoms, suicidal ideation, or other conditions that make decision‐making difficult, or if there are any safety concerns, individual consultations are conducted as the default.

**Figure 1:**
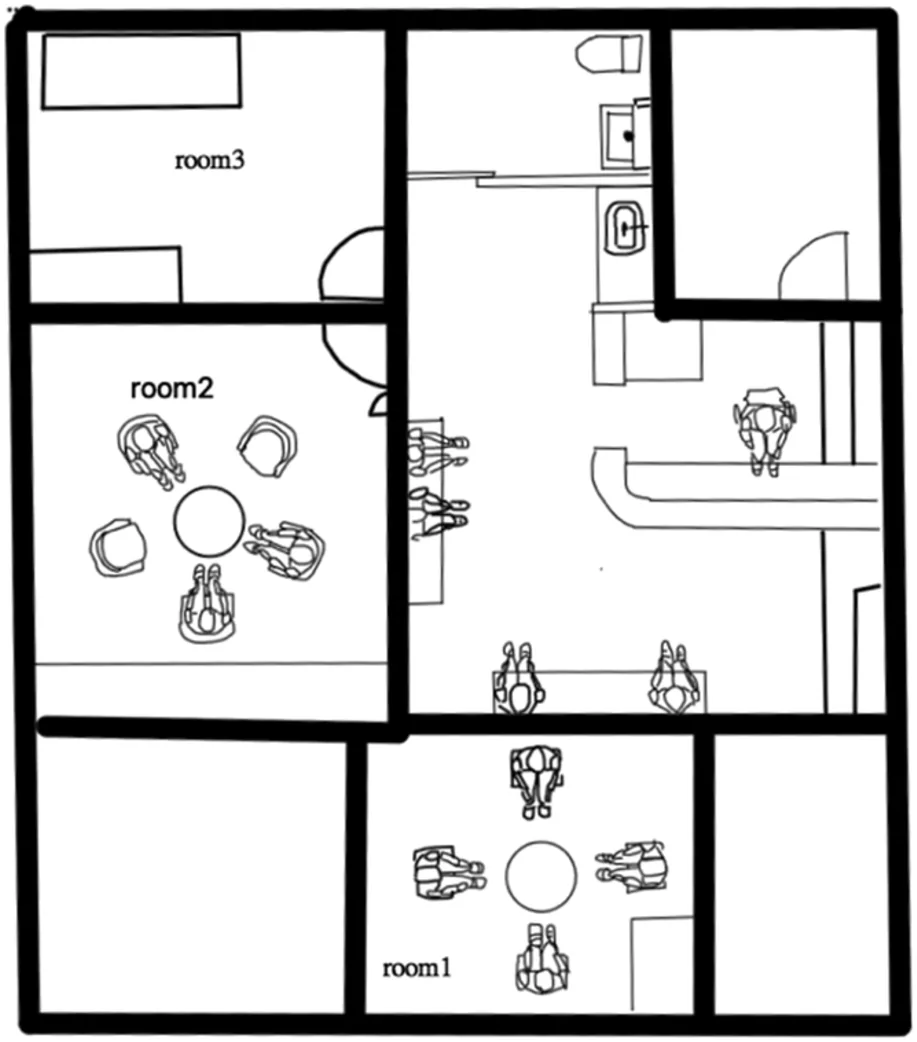
Layout of the consultation room. Seating arrangement of clinicians and the client at Yuurin Clinic.

Figure 1 illustrates the layout of the consultation room, depicting the typical seating arrangement used during the sessions.

#### Reflecting consultations

If a client opted for a reflecting consultation, a psychiatrist (the first author), who was trained in OD at Yuurin Clinic, and a family therapist who completed the “Dialogical approaches in couple and family therapy ‐ Psychotherapy Trainers Training” in Finland, the second author, conducted the consultation together. The standard structure involved the client, the family therapist, and the psychiatrist. However, if the client temporarily preferred an individual consultation, such as during periods of poor physical condition, the consultation was conducted solely by the psychiatrist.

Depending on the situation, external supporters such as visiting nurses and/or mental health professionals from Yuurin Clinic—including psychiatric social workers and nurses—could also participate. If external supporters joined, it was based on the client's request or a request from the supporters, with the client's consent. Similarly, internal professionals participated when the psychiatrist deemed necessary, when requested by the client, or when internal professionals already involved in the case requested a joint consultation, with the client's consent.

As a general rule, consultations were held in Room 2, and Room 1 was used if Room 2 was occupied. Clients were free to choose their seating upon entering, and clinicians sat around a round low table (see Figure 1).

Reflecting consultations were approximately one hour in length but the duration was adjusted based on the client's condition or preference. Sessions began with a psychiatrist and a family therapist facing the client (and any family/supporters present), asking an open‐ended question such as, “Is there anything you would like to talk about today?” Rather than being guided by the clinicians, the client spoke freely about what they wished, and clinicians responded to any questions from the client as needed.

When the client or the supporter reached a natural pause, the psychiatrist or the family therapist announced the start of the reflecting process. Before starting, the client was informed that he or she was free to ignore the reflecting dialogue to avoid it being perceived as indirect advice. During the reflecting process, the psychiatrist and the family therapist turned away from the client and spoke to each other.

After reflecting, they turned back to the client and asked, “If there is anything that has come to mind and you would like to share, please feel free to do so.” Reflecting was generally done once or twice per session and was completed at least five minutes before the end. If suicidal ideation was mentioned, the psychiatrist respectfully asked to explore it further and, if needed, directly proposed treatment adjustments or a switch to individual consultation. If medically necessary issues, such as side effect checks, were not addressed in conversation, they were asked by the psychiatrist before closing the session.

The psychiatrist and the family therapist followed these principles in reflecting:

1. Reflect only on the content of the current conversation.
2. Do not bring in external contexts (e.g., diagnoses not mentioned).
3. Speak in tentative opinions, not definitive interpretations or instructions.
4. Do not consolidate opinions into one.
5. Avoid negative comments or excessive praise about the client.
6. During reflecting, do not look at the client or communicate nonverbally.

#### Individual Consultations

Similar to reflecting consultations, individual consultations were held in Room 2 when available, and in Room 1 when Room 2 was occupied (see Figure 1). However, they differed in consultation length, structure, and the absence of reflecting dialogue. The client chose their seat freely, and the psychiatrist sat around the round table. The psychiatrist alone conducted the consultation, which typically lasted between 5 and 20 min.

### Study Design

This study was an observational investigation conducted at Yuurin Clinic, where medical consultations were provided as described above. The aim of the study was to examine whether the tense of the client's utterances during the conversations changes before and after reflecting.

Allocation to either the reflecting consultation or the individual consultation was based on client preference. Because of this selection bias, the individual consultation group cannot be regarded as a control group. Thus, statistical comparisons between the two groups were not performed. The data of individual consultation were presented for a reference purpose only.

### Participants

Clients receiving treatment at Yuurin Clinic between January 2024 and January 2025 were eligible for participation. Inclusion criteria were being 18 years of age or older and undergoing treatment for a mental disorder diagnosed according to the DSM‐5‐TR (Diagnostic and Statistical Manual of Mental Disorders, Fifth Edition, Text Revision). Written and verbal informed consent was obtained from all participants. In cases where individuals other than the client (e.g., family members or external supporters) were present during the consultation, consent was obtained from all participants. If even one person declined, the session was not recorded and excluded from the study. Written informed consent was obtained from all participants for their participation in this study.

Consent was not obtained at the beginning of the initial consultation but obtained after the first session and within one year of the initial visit. The recordings were performed at the next session after informed consent.

Among the 18 cases in the reflecting consultation group to whom the physician explained the study and asked to give informed consent, one case discontinued visits after consent was obtained, and seven cases did not provide consent. Thus, 10 cases were included in the analysis. In one of these cases, a married couple—both with psychiatric diagnoses—attended together and both provided consent; however, only the wife, who had originally requested the consultation, was included in the data analysis. In another case, one external supporter was present; although consent was obtained from the supporter and the entire session was recorded, only the client's speech data were used for analysis. In a separate case, a visiting nurse from Yuurin Clinic was also present during the session as part of the clinical team. Although the session was recorded in full, only the client's speech data were included in the analysis.

Among the 10 cases in the individual consultation group to whom the psychiatrist explained the study and asked to give informed consent, one discontinued visits after consent was obtained, and five declined consent, leaving four cases for analysis.

The final sample included 10 clients in the reflecting consultation group and four clients in the individual consultation group. The reflecting consultation group consisted of four males and six females, with a mean age of 44.6 years (SD = 12.9; range, 27–61 years). Their diagnostic categories, based on DSM‐5‐TR codes, were F2 in one client, F3 in four clients, and F4 in five clients. The individual consultation group consisted of four clients, all of whom were male, with a mean age of 47.8 years (SD = 14.0; range, 25–63 years). Their diagnostic categories, based on DSM‐5‐TR codes, were F2 in one client, F3 in one client, and F4 in two clients.

This study was approved by the Research Ethics Committee of the Juntendo University Faculty of Medicine.

### Data collection

Recording was started simultaneously with the beginning of each consultation and ended before the conversation to schedule the next appointment began. The recording was conducted by placing the recording device on the table so that the client's voice could be captured as clearly as possible.

The audio data were transcribed using a speech recognition program (programming language: Python 3.10; speech recognition package: Vosk 0.3.45; speech recognition model: vosk‐model‐ja‐0.22). The author then checked the recording, corrected the transcription as needed, and a co‐researcher performed a double‐check, producing the final textual data. During transcription, proper nouns were anonymized so that individuals could not be identified, and all audio data were processed offline.

When handling research‐related information, personal identifiers were removed, and the data were managed using research IDs.

### Data analysis and statistical methods

The tense of each utterance was classified basically according to the method established in previous studies.^13^, ^14^ Detailed methods are shown in the Supplementary Methods. In brief, transcription was conducted based on recordings of free conversation during the consultations. Using the anonymized transcribed text data, the utterances were classified based on their grammatical structure and the temporal reference point (present, past, recent past, or future), in order to determine whether each utterance referred to events in the present, past, recent past, or future. In this study, “recent past” was defined as the period between the previous and current consultation. This distinction was introduced because events occurring during this interval are often closely linked to the client's current clinical concerns in routine outpatient practice, whereas the author's clinical impression was that reflecting was often followed by references to more distant past experiences.

The segmentation units were determined through discussion between the psychiatrist (T.S.) and the family therapist (H.T.), both of whom were present during the session. Tense classification was then performed independently by two raters (T.S. and H.T.) using the same segmentation units. To assess inter‐rater reliability, Cohen's κ coefficients were calculated for both the original 15‐category classification and the four‐category classification. The final analyses were based on the classification by one rater (T.S.).

After the duration of each classified segment was measured, the proportion of time spent in each tense was calculated using the speaker's total speaking time as the denominator.

These proportions were calculated in two intervals: from the beginning of the consultation to the start of the first reflecting session, and from the end of the first reflecting session to the conclusion of the consultation or the start of the second reflecting session. For the primary analysis, the various subcategories were grouped into four major categories: future, present, recent past, and past. We examined whether the total proportion of speaking time in each of these four major categories changed before and after the reflecting session. The transcription data from the first recorded session were used for statistical analysis. For the individual consultation group, tense classification was conducted in the same manner. The session was divided into two halves using the midpoint as reference, and changes in the proportions of tense categories before and after that point were analyzed.

### Statistical Analysis

In this study, we examined how the proportions of tense usage (future, present, recent past, past) in the speaker's utterances changed before and after reflecting, using the transcription data from the first recorded consultation for both the reflecting consultation group and the individual consultation group.

To evaluate overall changes in tense distribution, a two‐way repeated measures ANOVA was conducted with tense (future, present, recent past, and past) and time (before and after reflecting) as within‐subject factors to examine their interaction. Mauchly's test of sphericity was performed, and when the assumption of sphericity was violated, Greenhouse–Geisser and Huynh–Feldt corrections were applied.

Furthermore, in the reflecting consultation group, Wilcoxon signed‐rank tests were conducted as post hoc analyses to examine changes in each tense category following the significant interaction observed in the repeated measures ANOVA. The significance level was set at *p* < 0.05.

## RESULTS

Cohen's *κ* coefficients were 0.849 for the original 15‐category classification (*p* < 0.001) and 0.914 for the four‐category classification (*p* < 0.001). The final results presented below are based on one rater's classification, while analyses based on the other rater's classification showed essentially similar patterns.

Future‐oriented utterances were not observed either before or after reflecting in any case. Therefore, future‐oriented utterances were excluded from the repeated‐measures ANOVA, which was conducted using the remaining three tense categories: past, recent past, and present. Mauchly's test of sphericity indicated that the assumption of sphericity was not violated for the time × tense interaction (*p* = 0.468). The repeated‐measures ANOVA showed a statistically significant time × tense interaction, F(2, 18) = 7.165, *p* = 0.005, partial *η* ^2^ = 0.443. In all cases, the proportion of utterances based on past‐oriented tense increased.

Following this significant interaction, Wilcoxon signed‐rank tests were conducted as post‐hoc analyses to examine changes in each tense category.

**Figure 2:**
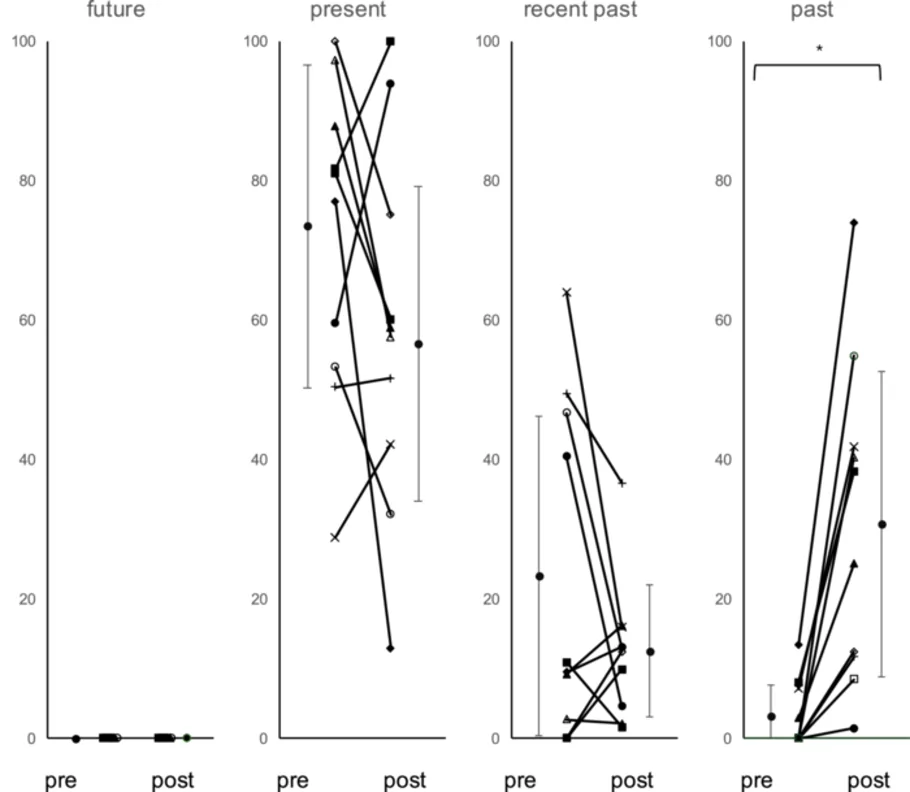
Changes in tense proportions pre‐ and post‐reflecting (reflecting consultations). Each line represents an individual case. Circles with vertical error bars indicate mean values and standard deviations. Mean proportions (%) of clients' utterances were: Future 0 → 0, Present 73.6 → 56.7 (*p* = 0.093), Recent past 23.3 → 12.5 (*p* = 0.241), and Past 3.1 → 30.8 (*p* = 0.005). “Pre” and “Post” refer to the segments before and after the reflecting talk, respectively. ^＊^ *p* = 0.005 by Wilcoxon signed‐rank tests.

As a result, the proportion of past‐oriented utterances significantly increased after reflecting (*z* = −2.803, *p* = 0.005, *r* = 0.89). No significant changes were observed in present‐ or recent‐past‐oriented utterances. Future‐oriented utterances were not observed either before or after reflecting (Figure 2).

Table 1 presents examples of utterances in the “Past” tense from clients' speech following reflecting, along with their corresponding subcategory classifications.

**Table 1:** Examples of utterances in the “Past” tense (Reflecting Group).

Categories	Example utterance	Interpretation
PF (Future from past)	No corresponding example was identified in the present dataset.	Future‐oriented utterance from a past reference point.
PN (Present from past)	“When I was a student, I was talking with the counselor, and biting my nails.”	A present state or behavior described from a specific point in the past
PP (Past from past)	“I had been working for over 10 years to rebuild that kind of relationship.”	A prolonged effort carried out before a specific point in the past

Although the sample size of the individual consultation group is too small to allow formal statistical comparison with the reflecting consultation group, a preliminary analysis by repeated measures ANOVA showed no significant interaction between tense and time. The corresponding data are provided for reference in Supplementary Figure 1.

## DISCUSSION

This study examined changes in the tense structure of clients' utterances before and after reflecting. Present‐oriented utterances accounted for the largest proportion both before and after reflecting, and this overall tendency did not appear to change substantially. Nevertheless, past‐oriented utterances increased after reflecting.

Although the number of subjects in the individual consultation group was too small to allow reliable statistical interpretation, descriptive analysis showed no major change in the overall distribution of tense in the individual consultation group.

It is possible that the clients experienced a sense of space that had not been present when receiving direct advice or instruction by observing clinicians discussing what the client had spoken. As a result, self‐reflection may have been more freely facilitated, potentially leading to the recall of past experiences.

Andersen, the originator of reflecting, stated that families tend to fall into a dualistic perspective when discussing problems, but that reflecting fosters multiple perspectives and understandings.^8^ The finding that clients more frequently recalled past experiences after reflecting might indicate that clients, who had previously been trapped in binary thinking, choosing between option a or b regarding their present problems, came to acquire a new perspective, option c, based on reflections on past experiences.

Andersen also argued that there are two types of human change. He described the first type as “comes from outside the person, from another, as a one‐sided instruction,” and identified the second type as “change comes from inside the person, as a result of an interplay with others”.^15^ This second type of change is not paternalistic but is rather associated with recovery. The emergence of inner awareness, including the recall of past experiences, can be considered one element of this type of change.

Garrido‐Fernández et al. reported that a couple therapy using reflecting for individuals with gambling addiction was associated with the development of positive self‐image and a complex self‐awareness profile,^16^ suggesting the potential of reflecting to promote self‐awareness.

Considering these previous studies, the present finding that the tense of the utterances during conversation changed after reflecting may be one aspect of the promotion of internal dialogue and deepening of self‐reflection. Reflecting may therefore have potential clinical value as a practice that supports self‐reflection in psychiatric care.

In this study, allocation was based on client preference, and the individual consultation group was not a formal control group. In addition, consultation duration differed substantially between the groups. Therefore, the observed increase in past‐oriented utterances cannot be attributed solely to reflecting itself. Furthermore, although the present findings may suggest that increased references to the past reflect enhanced self‐reflection, this pattern may also reflect narrative expansion, longer consultation duration, natural topic shifts, or the influence of the clinicians' own language during the reflecting dialogue. These findings should therefore be interpreted cautiously.

This study has many major additional limitations that should be considered in future studies. First, the sample size was small, and no definitive conclusions can be drawn. Thus, the present study should be treated as a preliminary, hypothesis‐generating study. Second, there was no matched control group. The individual consultation group was small and was not intended to serve as a statistical control group for direct comparison with the reflecting consultation group. Therefore, the main focus of the analysis was the within‐group change before and after reflecting in the reflecting consultation group, while the data from the individual consultation group were presented only as reference data. Although descriptive analysis showed no major change in tense distribution in the individual consultation group, this finding should be interpreted cautiously. In addition, the reflecting consultations were generally longer than individual consultations, which might have influenced the temporal structure of the dialogue. For future studies, a control group for which the group allocation was based on randomization, rather than client preference, should be studied. Third, although inter‐rater reliability for tense classification was assessed using Cohen's *κ*, the interpretation of the inter‐rater reliability values requires caution. To calculate Cohen's *κ*, both raters had to classify the same units of analysis. Therefore, the segmentation units were determined in advance through discussion between the two clinicians who participated in the consultations. The *κ* coefficients may therefore have been influenced by the use of these jointly determined segmentation units. In addition, the raters' shared clinical background may also have contributed to the high agreement. Future studies should assess inter‐rater reliability using raters who are not directly involved in the consultations. AI‐assisted assessment of tense may be explored as a possible future approach. Additionally, as this study analyzed only the first recorded session, longitudinal changes over the course of treatment remain unexplored.

Although this was a preliminary observational study aiming to generate hypotheses rather than provide definitive conclusions, these initial findings may provide a basis for planning larger‐scale future research.

Finally, reflecting is not simply a unidirectional intervention by clinicians; rather, mutual observation and mutual transformation between clinicians and clients are considered essential components. However, the present study focused solely on client outcomes. Future studies should also examine changes in the therapist processes, including clinicians' reflections and evaluations, as well as feedback from clients, to gain a more comprehensive understanding of the mutual effects of the reflecting process. Furthermore, as reflecting may invite the recall of experiences, clinicians should remain mindful of clients' readiness and of the possibility that difficult memories may emerge.

## CONCLUSION

This study examined the changes in the tense of the utterances of a client during conversations before and after reflecting in OD settings. The results indicated a significant increase in the client's use of the past tense following reflecting. These findings suggest that reflecting may facilitate the recall of past experiences and promote self‐reflection.

Compared to OD, which is difficult to directly implement in Japan due to differences in medical systems, reflecting offers a more flexible approach and holds promise for wider adoption. However, as Andersen cautioned, it is important that reflecting not be reduced to a mere technique; rather, it should be shared and practiced in a manner that preserves its philosophical underpinnings.

## AUTHOR CONTRIBUTIONS

**Tomoyuki Sugita:** Conceptualization; methodology; participation in the reflecting consultations as the psychiatrist; data collection, transcription, tense categorization; formal analysis; writing—original draft. **Hiromi Tsujii:** Participation in the reflecting consultations as the family therapist, methodology; contribution to determining the segmentation units; independent assessment of tense categorization. **Suimei Morikawa:** Supervision; critical review of the manuscript. **Takao Iwanami:** Supervision: final approval of the manuscript. **Tadafumi Kato:** Conceptualization; study design; methodology; supervision; guidance in data visualization (tables and figures); critical revision of the manuscript for important intellectual content.

## CONFLICT OF INTEREST STATEMENT

The last author (Tadafumi Kato) is an Editorial Board member of Psychiatry and Clinical Neurosciences Reports. He is not involved in the editorial decision‐making of this article. Other authors declare no conflicts of interest.

## ETHICS STATEMENT

This study was approved by the Research Ethics Committee of the Juntendo University Faculty of Medicine.

## PATIENT CONSENT STATEMENT

Written and verbal informed consent was obtained from all participants involved in the recorded consultations. Additional consent for publication was obtained for the anonymized material presented in the figures.

## CLINICAL TRIAL REGISTRATION

Not applicable, as this was an observational study conducted within routine clinical consultations.

## Supporting information

Supplementary Figure 1. Changes in Tense Proportions During the First and Second Halves of the Session (Individual Consultations). Each line represents an individual case. Circles with vertical error bars indicate mean values and standard deviations. Mean proportions (%) of clients' utterances were: Future 0 → 0, Present 71.5 → 69.9, Recent past 27.2 → 18.6, and Past 1.32 → 11.5. “Pre” refers to the first half of the individual consultation session, and “Post” refers to the second half.

Supplementary methods revised.

## Data Availability

The data that support the findings of this study are available on request from the corresponding author. The data are not publicly available due to privacy or ethical restrictions.
