# Supplementary material for: Possible influence of reflecting on the tense of the client's utterances: A preliminary finding
Source: PCN Rep. 2026 Sep 7;5(3):e70407. doi: 10.1002/pcn5.70407 (PMC13549221; doi:10.1002/pcn5.70407)
Supplement: Supplementary file 1 [file PCN5-5-e70407-s001.pdf]

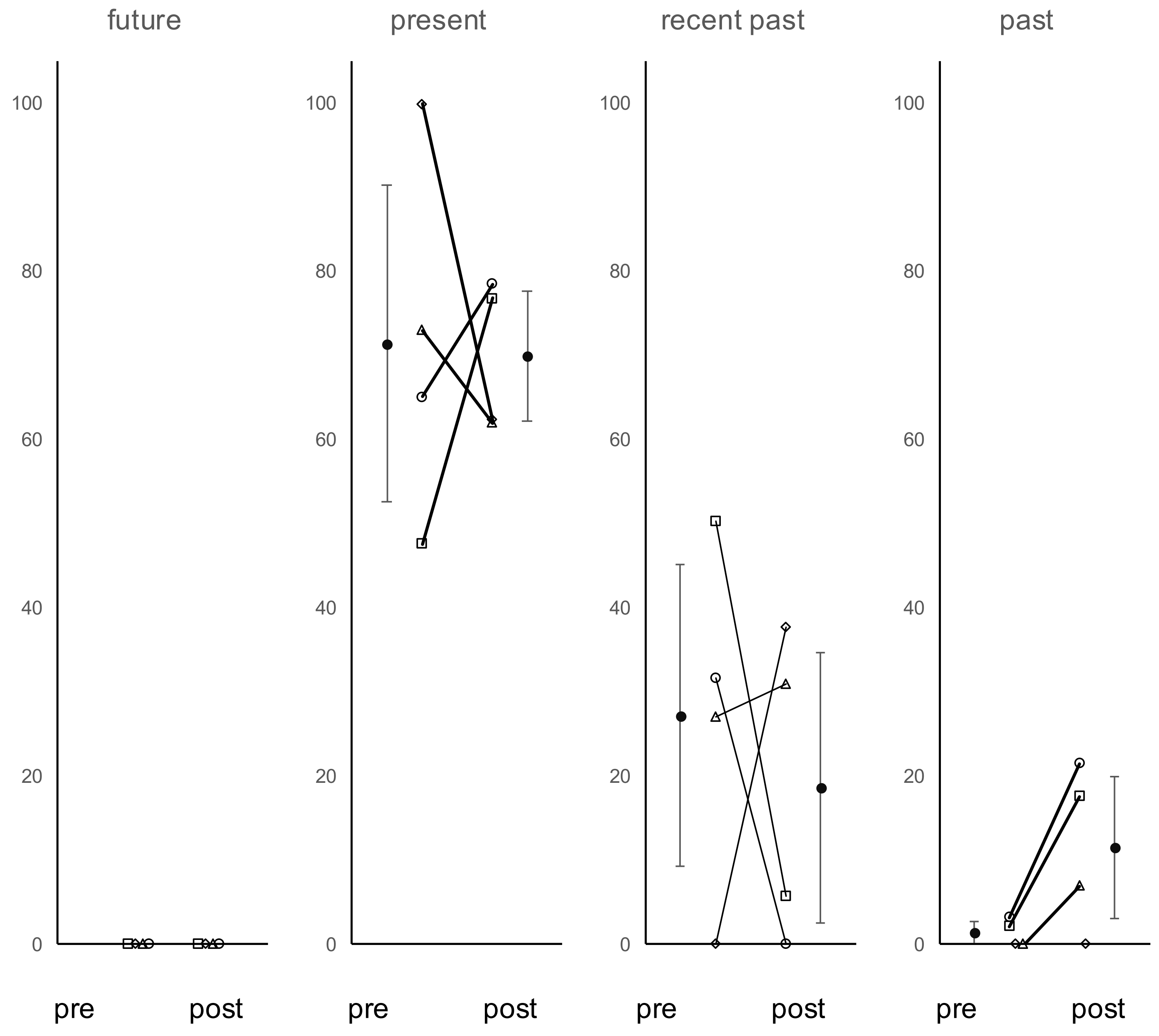
future
present
recent past
past
100
80
60
40
20
0
pre
post
