# Supplementary material for: Possible influence of reflecting on the tense of the client's utterances: A preliminary finding
Source: PCN Rep. 2026 Sep 7;5(3):e70407. doi: 10.1002/pcn5.70407 (PMC13549221; doi:10.1002/pcn5.70407)
Supplement: Supplementary file 2 [file PCN5-5-e70407-s002.pdf]

Supplementary methods

The tense of each utterance was classified primarily based on the methods established in previous studies (1, 2). In these studies, the following nine tense categories were used: FF (future from a point in the future: 1), FN (present at a point in the future: 2), FP (past from a point in the future: 3), NF (future from the present: 4), NN (present: 5), NP (past from the present: 6), PF (future from a point in the past: 7), PN (present at a point in the past: 8), and PP (past from a point in the past: 9). In contrast to the conversation settings used in these previous studies, however, the main topics in clinical consultations tend to concern events occurring between the previous and the current sessions. Therefore, in addition to the nine tense categories used in the previous studies, we defined the time between the previous and current consultations as “recent past” and added six new categories: FP″ (recent past from a point in the future: 10), NP″ (recent past from the present: 11), P″F (future from a point in the recent past: 12), P″N (present at a point in the recent past: 13), P″P″ (recent past from a point in the recent past: 14), and P″P (past from a point in the recent past: 15), resulting in a total of 15 categories. The 15 categories were further grouped into four major categories: future (FF, FN, FP″, FP), present (NF, NN, NP″, NP), recent past (P″F, P″N, P″P″, P″P), and past (PF, PN, PP).

Based on the text data, one of the above tense categories was assigned to each narrative segment in which the content was judged to remain consistent. Even within a single sentence, the category was reassigned if the content was judged to have changed. The segmentation units were determined by the consensus between the psychiatrist (T.S.) and the family therapist (H.T.) who were present during the session.

The tense assignment was performed independently by two raters (T.S. and H.T.). To assess inter-rater reliability, Cohen's κ coefficients were calculated for both the original 15-category classification and the four-category classification.

Cohen's κ coefficients were 0.849 for the original 15-category classification ($p < 0.001$) and 0.914 for the four-category classification ($p < 0.001$). The final results presented below are based on one rater's classification, while analyses based on the other rater's classification showed essentially similar patterns.

We examined whether the total proportion of speaking time in each of four major categories changed before and after the reflecting session. The transcription data from the first recorded session were used for statistical analysis.

References

1. Onoda K, Otake M. Kenkō chōju kōrei-sha no kaiwa ni okeru genzai kako mirai no wariai to sui-i (Balance and Trend Analysis of Present, Past and Future tenses in Conversation among Healthy Older Adults). Journal of Artificial Intelligence Society (Published by Japanese Society for Artificial Intelligence). 2014;SIG-SKL-19-01.

2. Onoda K, Otake M. Estimation of Mental Time by Analysis of Tense During Conversation. Ambient Intelligence for Health and Cognitive Enhancement: Papers from the 2015 AAAI Spring Symposium; Stanford University, California, USA: Association for the Advancement of Artificial Intelligence (AAAI); 2015. p. 55–61.
